# Synthetic Aspects and Electro-Optical Properties of Fluorinated Arylenevinylenes for Luminescence and Photovoltaics

**DOI:** 10.3390/ma6041205

**Published:** 2013-03-25

**Authors:** Carmela Martinelli, Gianluca M. Farinola, Vita Pinto, Antonio Cardone

**Affiliations:** 1 Istituto di Chimica dei Composti OrganoMetallici CNR-ICCOM di Bari, Via Orabona 4, Bari I-70125, Italy; E-Mail: martinelli.carmela@chimica.uniba.it; 2 Deparment of Chemistry, Università degli Studi di Bari “Aldo Moro”, Via Orabona 4, Bari I-70125, Italy; E-Mails: gianlucamaria.farinola@uniba.it (G.M.F.); vita.pinto@uniba.it (V.P.)

**Keywords:** arylenevinylenes, fluorinated materials, Stille cross-coupling, organic semiconductors, conjugated materials

## Abstract

In this review, the main synthetic aspects and properties of fluorinated arylenevinylene compounds, both oligomers and polymers, are summarized and analyzed. Starting from vinyl organotin derivatives and aryl halides, the Stille cross-coupling reaction has been successfully applied as a versatile synthetic protocol to prepare a wide series of π-conjugated compounds, selectively fluorinated on the aromatic and/or vinylene units. The impact of fluoro-functionalization on properties, the solid state organization and intermolecular interactions of the synthesized compounds are discussed, also in comparison with the non-fluorinated counterparts. Luminescent and photovoltaic applications are also discussed, highlighting the role of fluorine on the performance of devices.

## 1. Introduction

Current interest in the emerging fields of plastic electronics and photonics is based on the peculiar optical and semiconducting properties of organic π-conjugated materials, which can be designed and developed for applications in many opto-electronic devices [1,2,3], such as organic light-emitting diodes (OLEDs) [4,5,6,7] organic thin film transistors (OTFTs) [8,9], sensors [10] and photovoltaics [11,12,13]. The key advantages of organic semiconductors with respect to their inorganic counterparts reside in the possibility to fine-tailor properties on the molecular scale (e.g., band gap, HOMO and LUMO energy levels, charge mobility, thermal and chemical stability, processability) by proper design of the conjugated skeleton and functionalization, thus enabling the use of low-cost technologies in the fabrication of organic-based devices.

From a synthetic point of view, the functionalization of organic compounds with appropriate substituents plays a key role in the control and tuning of structural and chemico-physical properties. As a functional group, fluorine possesses a unique and extraordinary capability to modify the properties of the materials [14,15,16] through various effects. Deep investigations on these features often reveal even more interesting and unexpected results. The main effects of fluorine are related to the special characteristics of this small atom, such as the highest electronegativity (in the Pauling scale, EN = 4) and the high C–F bond energy (about 480 KJ/mol) in the periodic table. The strength of the C–F bond improves the thermal and chemical stability of fluorinated organic compounds, which can result in an enhancement of the devices life-time. The electron-withdrawing effect of the fluorine atoms causes a lowering of the HOMO and LUMO energy levels of organic semiconductors, facilitating electron injection from metal electrodes and improving negative charge transport capability. As a consequence, fluorinated compounds behave as ambipolar or n-type semiconductors and can find useful application as active materials in bipolar or n-type OTFT [17,18,19,20,21,22]. Furthermore, due to the strong polarization of the C–F bond, F····H–C H-bonds occur in organic fluorinated materials [23], which can affect in a significant way molecular geometry and the charge distribution of the molecules, playing an interesting role in the solid state organization. In light of these considerations, the fluoro-functionalization has been explored as a chemical and structural modification in several classes of organic materials. In this review, we describe our contribution in this field, through the synthesis and electro-optical investigation of arylenevinylene compounds for application in luminescent devices and photovoltaics.

## 2. Results and Discussion

### 2.1. Fluorinated Oligo(arylenevinylene)s

#### 2.1.1. Synthesis

Although the initial interest for conjugated oligomers was related to their possible use as model compounds, aiming to investigate the properties of corresponding polymeric materials [24], very shortly, they themselves have become very attractive as active materials for electro-optical applications, showing, in some cases, even better performances with respect to their polydisperse homologs [25,26,27,28,29].

**Scheme 1:**
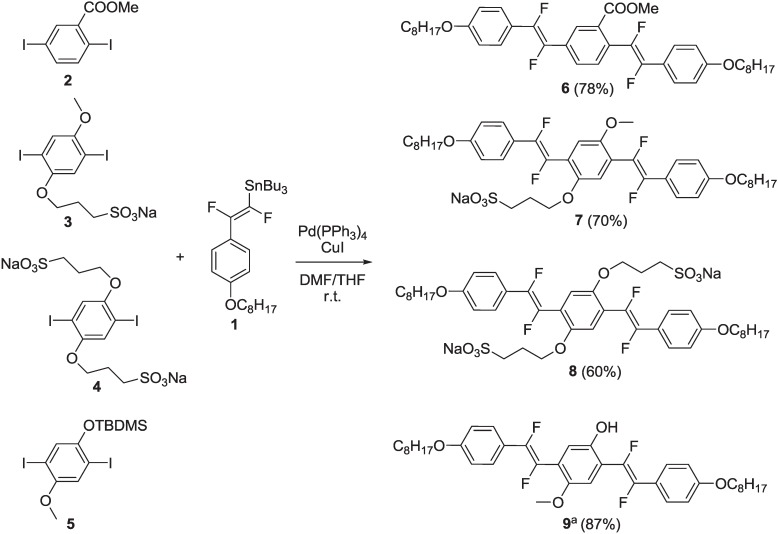
Synthesis of fluorinated oligomers **6**–**9**. ^a^ *In situ* hydrolysis by 40% aq KF.

Recently, we have developed a versatile and stereoselective synthetic protocol to prepare all *trans* oligoarylenevinylenes fluoro-functionalized on the vinylene units, based on the Stille cross-coupling reaction. Additional polar and non-polar groups have been introduced onto aromatic rings in order to modulate the solubility of the final compounds in different solvents [30]. A series of oligomers (**6**–**9**) was prepared by reacting (*E*)-1-2-difluoro-2-[4-(octyloxy)phenyl]vinyl-1-tributilstannane **1** with diiodoaryls **2**–**5** in the presence of Pd(PPh_3_)_4_ as the catalyst, CuI in a stoichiometric amount, DMF/THF as the reaction solvent, overnight at room temperature (Scheme 1).

**Scheme 2:**
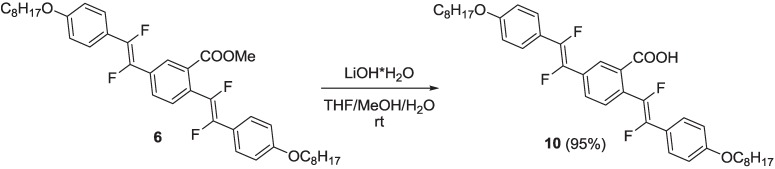
Synthesis of fluorinated oligomer **10**.

The Stille reaction proceeds regio- and stereo-selectively, affording oligoarylenevinylenes **6**–**9** with all-*trans* vinylene units. Compound **9** was obtained after *in situ* hydrolysis with 40% aq KF. Oligomers **6**, **7** and **9** were purified by chromatography and obtained in good yields as a yellow solid, a dark orange solid and a pale yellow solid, respectively. Oligomer **8** was purified by several crystallizations from DMF/acetone and obtained pure as a grey solid. The alkoxy chains bonded to the terminal phenyl rings confer a good solubility to compounds **6**, **7** and **9** in organic solvent, such as CHCl_3_, CH_2_Cl_2_, MeOH, while compound **8** shows good solubility only in aprotic dipolar solvents, such as DMF and DMSO. Then, compound **6** was submitted to a basic hydrolysis with LiOH·H_2_O in THF, MeOH and H_2_O to yield oligomer **10**, bearing a free carboxylic group (Scheme 2).

**Scheme 3:**
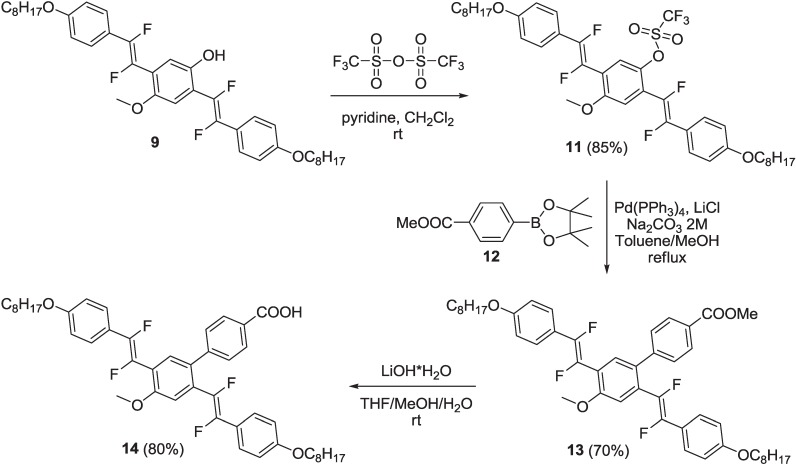
Synthesis of fluorinated oligomer **14**.

Compound **9** was converted in triflate derivative **11**, which, in turn, reacted with commercial 4-(4,4,5,5-tetramethyl-1,3,2-dioxaborolan-2-yl)benzoate, **12**, under the Suzuki reaction conditions, to yield intermediate **13**. Finally, compound **13** was submitted to a basic hydrolysis, under the same conditions of **10**, to yield oligomer **14** with the free carboxylic unit (Scheme 3).

**Scheme 4:**
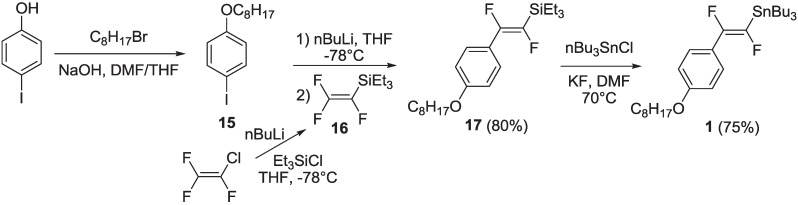
Synthesis of organostannane **1**.

The synthesis of intermediate (*E*)-1,2-difluoro-2-[4-(octyloxy)phenyl]vinyl-1-tributilstannane **1** was carried out by extending a literature procedure [31], as shown in Scheme 4.

1-Iodo-4-(octyloxy)benzene **15** (obtained by alkylation of commercial 4-iodophenol) was lithiated by nBuLi, then reacted with triethyl(trifluorovinyl)silane **16** (prepared by lithiation of commercial chlorotrifluoroethene with nBuLi followed by quenching with chlorotriethylsilane—see Scheme 7 and related discussion), to yield intermediate vinyl silane **17**. This, in turn, was submitted to a transmetallation process with nBu_3_SnCl in the presence of anhydrous KF in dry DMF and at 70 °C, to yield the organostannane **1**.

**Scheme 5:**
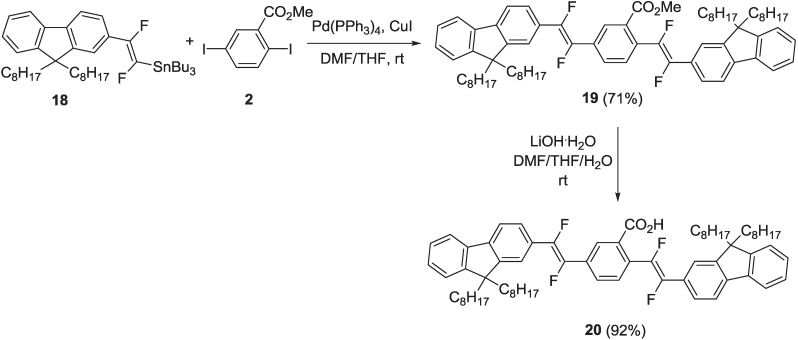
Synthesis of fluorinated oligomer **20**.

Fluorenevinylene oligomer (**20**) was also prepared following the same synthetic protocol adopted for the preparation of oligomers **6**–**9**, starting from organostannane **18** (Scheme 5).

**Scheme 6:**
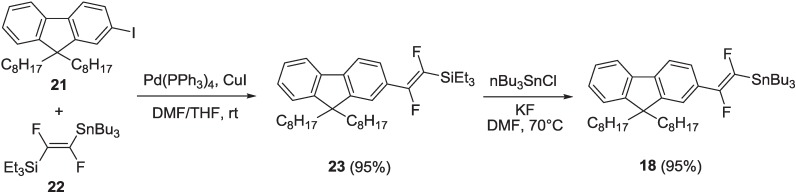
Synthesis of organostannane **18**.

Organostannane **18** reacted with diiodoaryl **2** in the Stille reaction conditions, to yield intermediate compound **19**. This was finally submitted to a basic hydrolysis with LiOH·H_2_O in THF, MeOH and H_2_O, to yield the final oligomer, **20**, with the free carboxylic unit. For the preparation of organostannane **18**, a different synthetic pathway was followed with respect to organostannane **1**, since the lithium derivative of 2-iodo-9,9-dioctylfluorene **21** (Scheme 6), obtained after its treatment with nBuLi, did not react with triethyl(trifluorovinyl)silane **16**. Therefore, intermediate **21** was coupled with (*E*)-[1,2-difluoro-(2-tributylstannyl)vinyl]triethylsilane **22** under the Stille reaction conditions, in the presence of Pd(PPh_3_)_4_ and CuI in DMF/THF, to yield compound **23** (Scheme 6).

Vinylsilane **23** was then submitted to a transmetallation reaction with nBu_3_SnCl and anhydrous KF in dry DMF at 70 °C, to yield organostannane **18**.

**Scheme 7:**
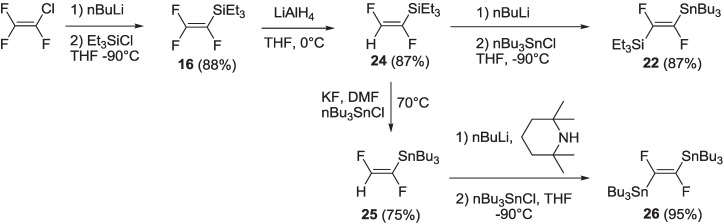
Synthesis of intermediates **22** and **26**.

Compound **22**, which can be used as a versatile organometallic building block in the preparation of asymmetrically 1,2-disubstitued difluorovinylene compounds, was synthesized for the first time in our laboratories following a synthetic protocol, which allows for preparation of two different organometallic reagents, (*E*)-[1,2-difluoro-(2-tributylstannyl)vinyl]triethylsilane **22** and (*E*)-1,2-difluoro-1,2-bis(tributylstannyl)ethene **26** [30] (Scheme 7).

Following a modified literature procedure [32], commercial chlorotrifluoroethene was lithiated by nBuLi and quenched with chlorotriethylsilane to yield intermediate **16**. Compound **16** was selectively hydrogenated with LiAlH_4_ at 0 °C to yield difluorovinylsilane **24**, which can be used as a starting reagent to synthesize compounds **22** or **26**. The use of chlorotriethylsilane instead of chlorotrimethylsilane was introduced in order to increase the boiling point of intermediate compounds, **16** and **24**, which can be, thus, easily separated from the reaction solvent by distillation. The same purification was not possible when chlorotrimethylsilane was used, because the trimethylsilyl derivatives corresponding to **16** and **24** have low boiling points very close to that of the reaction solvent. Treatment of intermediate **24** with nBuLi and nBu_3_SnCl afforded the organometallic compound, **22**. Treatment of intermediate **24** with anhydrous KF and nBu_3_SnCl in dry DMF at 70 °C, resulted in a transmetallation process to yield difluorovinyltributylstannane **25**. This was successively lithiated with lithium 2,2,6,6-tetramethylpiperide (obtained treating 2,2,6,6-tetramethylpiperidine with nBuLi) and quenched with nBu_3_SnCl, to yield bis-stannane **26**. All intermediate compounds and final products in Scheme 7 are easily purified by distillation.

**Scheme 8:**
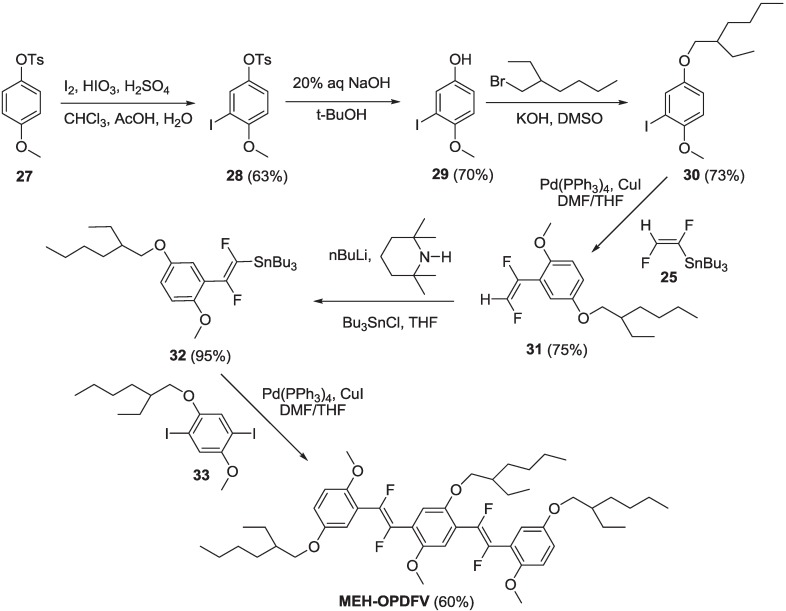
Synthesis of fluorinated oligomer **MEH-OPDFV**.

Taking advantage from organostannane **25**, we set up a synthetic sequence to prepare compound **MEH-OPDFV**, the oligomer model of **MEH-PPDFV**, as reported in Scheme 8.

Compound **27** was selectively iodinated in the *ortho*-position with respect to the methoxy group by iodine, iodic acid and sulfuric acid in a mixture of chloroform, acetic acid and water as the solvent, to yield compound **28**. Then, the tosylate group was removed by 20% aq sodium hydroxide in t-BuOH, giving compound **29**. Compound **29** reacted with 1-bromo-2-ethylhexane in the presence of KOH in DMSO to yield intermediate **30**. Compound **30** reacted, in the Stille conditions, with organostannane **25** to yield intermediate **31**. That was, in turn, converted in organostannane intermediate **32** by treatment with lithium 2,2,6,6-tetramethylpiperidide (prepared *in situ*) followed by quenching with nBu_3_SnCl. Finally, intermediate **32** was coupled in the Stille reaction with diiodo derivative **33**, to yield oligomer **MEH-OPDFV**, which was purified by chromatography and obtained as a colorless dense liquid.

#### 2.1.2. Spectroscopic Properties of Oligo(arylenedifluorovinylene)s

**Figure 1:**
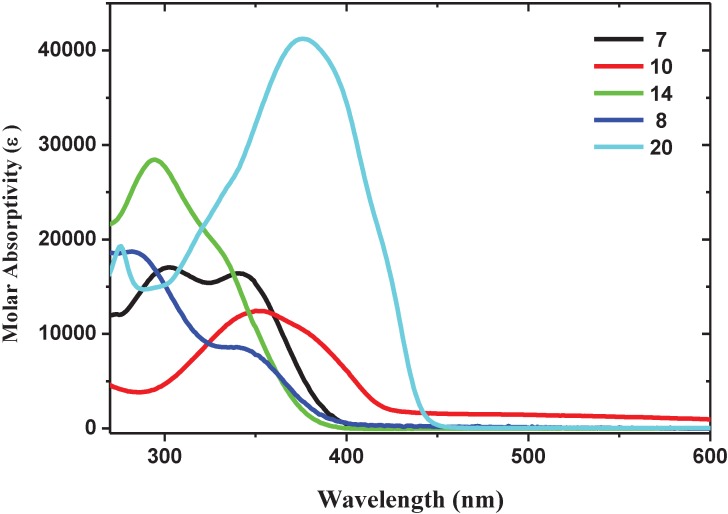
Absorption spectra of the oligomers in aerated solvents: **7**, **14** and **20** were measured in CH_2_Cl_2_, **8** in DMSO and **10** in MeOH.

UV-Vis spectra of the synthesized oligomers are shown in Figure 1.

All oligomers show absorption peaks in the range 270–400 nm. Oligomer **20** shows the highest molar absorptivity coefficient, ε, with a spectrum shifted at a longer wavelength with respect to the others oligomers. The absorption peaks are assigned to π–π* transitions along the conjugated backbone, and the ε values at λ_max_ range from about 14,000 to 20,000 M^−1^ cm^−1^ for **7**, **8** and **10**, reaching about 30,000 M^−1^ cm^−1^ for **14** and 40,000 M^−1^ cm^−1^ for **20** (Table 1).

**Table 1:** Photophysical properties of oligomers. Emission quantum yields Φ in solution were measured using quinine sulfate in 0.5 M H_2_SO_4_ as the standard [33].

Oligomer	Molar Absorptivity ε (×10^3^ M^−1^ cm^−1^) (λ, nm)	Emission λ_max_ (nm)	Φ (%) (solvent)
7	13 (302), 12 (342)	436	0.87 (CH2Cl_2_)
8	14 (283), 6 (342)	440	0.97 (DMSO)
10	14 (352)	466	5.4 (MeOH)
14	29 (295)	458	0.43 (CH_2_Cl_2_)
20	19 (276), 41 (376)	444, 471	8.12 (CH_2_Cl_2_)

**Figure 2:**
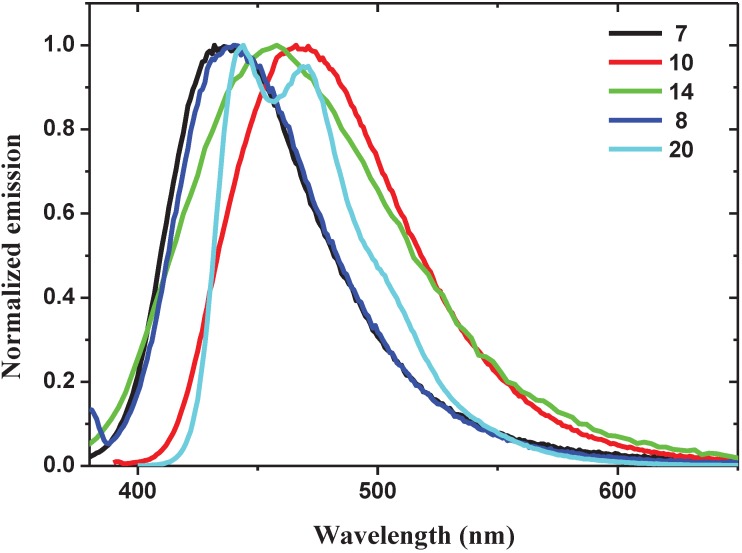
Normalized emission spectra of the oligomers in aerated solvents: **7**, **14** and **20** were measured in CH_2_Cl_2_, **8** in DMSO and **10** in MeOH.

All oligomers are luminescent in solution with maxima peaks in the blue and blue-green region of the visible spectrum (Table 1, Figure 2). Oligomers **10** and **20** show the higher emission quantum yields. Oligomers **7** and **8** show similar emission profiles (λ_max_ at 436 and 440 nm, respectively), they are slightly blue shifted with respect to those of **10**, **14** and **20**. These experimental observations are in accordance with theoretical calculations that assign the blue-shift to the torsional distortion of conjugated backbone induced by repulsive intramolecular interactions between fluorine atoms on the vinylene units and oxygen atoms of the alkoxy groups in the *ortho*-position on the aromatic rings [34].

Oligomers **7**, **8**, **10** and **14** have the same main conjugated backbone, the only difference being in the functional groups bonded to the central aromatic unit. In oligomers **7** and **8**, two alkoxy chains are bonded to the central phenyl unit. In oligomer **14**, one of the two alkoxy groups is substituted by a phenyl unit bearing an electron-withdrawing carboxylic group, causing a broadening and a red shift (458 nm) of the emission. By substituting both alkoxy groups of the central phenyl unit with a single electron-withdrawing carboxylic group in oligomer **10**, a further red shift (466 nm) in the emission is observed. Finally, oligomer **20**, consisting of two fluorenedifluorovinylene units bonded to a central phenyl ring bearing a carboxylic group, shows two emission peaks at 444 and 471 nm and a shoulder at 500 nm, with a typical profile of fluorene-based materials.

#### 2.1.3. Application of Oligomer **7** in a Chemical Sensor for Herbicide

**Figure 3:**
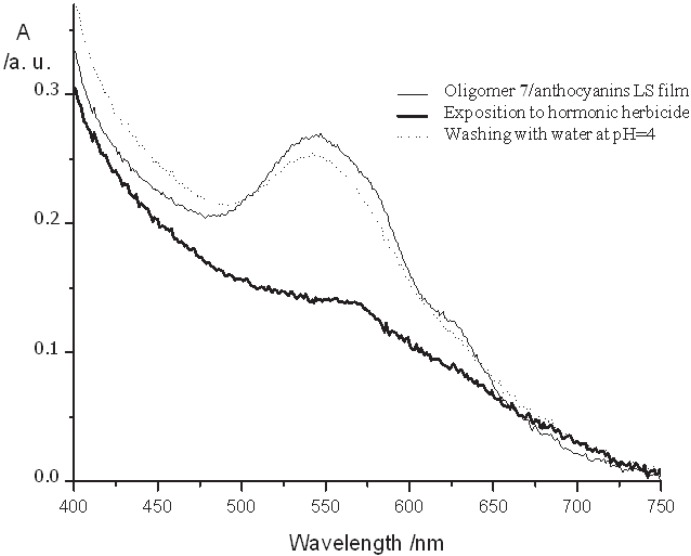
Variation of the visible spectrum of the LS sample, oligomer **7**/anthocyanins, during exposure to a solution of a commercial hormone type pesticide (Erbitox E30). Reproduced with permission from [35]. Copyright 2012 Elsevier.

The arylenevinylene structure, containing fluorinated double bonds, alkoxy end chains and a propyloxy sulfonate group, confers to oligomer **7** an amphiphilic behavior that is an essential prerequisite for Langmuir Schaefer (LS) transfer. For this reason, it was chosen as the conjugated anionic counterpart of positively charged hydrophilic anthocyanins in the deposition of thin films using the LS approach [35]. Anthocyanins are well-known pH indicators, with the absorption maximum that shifts from red in acid environment towards blue at basic pH, as a result of chemical structure variation [36]. Unfortunately, low stability prevents the use of anthocyanins pigments as active matrix in electronic devices, such as biosensors or dye sensitize solar cells. A number of physical and environmental factors affect anthocyanin stability, including temperature (the degradation rate increases with rising temperatures), UV light and pH. In general, low pH values stabilize anthocyanins, while basic pH values can cause a rapid degradation. The hydrophilic nature of anthocyanins prevent their transfer by the LS method, even though the positive charge on the anthocyanins allows a supramolecular assembling with negatively charged species in a dyad system suitable for LS technique. Taking oligomer **7** as the anionic counterpart of anthocyanins (from *Vitis vinifera*), the electrostatic interaction between the two compounds has been exploited to obtain a stabile supramolecular dyad for film fabrication with the LS method. Experimental results confirm, in fact, that the strong electrostatic interaction existing between anthocyanins and oligomer **7** is able to prevent the aggregation of the former compound in water medium and stabilizes the LS film. In addition, LS films of the dyad oligomer **7**/anthocyanins showed, also, an interesting increased stability of anthocyanins to UV exposure, with oligomer **7** acting as a protecting screen towards the UV radiation. Then, LS films of the oligomer **7**/anthocyanins dyad were investigated as active matrix for herbicide sensors (Figure 3).

Absorption spectra of the LS film oligomer **7**/anthocyanins deposited on a quartz substrate and incorporated in a home-made flow cell were acquired: the results showed a red shift and an almost complete bleaching of the absorption peak after exposition to hormonic herbicide, and the sensing process was completely reversible, disclosing the composite film as a potential colorimetric chemical sensor for the detection of a commercial herbicide.

### 2.2. Fluorinated Poly(arylenevinylene)s

#### 2.2.1. Synthesis of Poly(arylenevinylene)s Fluorinated on the Aromatic Ring

**Scheme 9:**
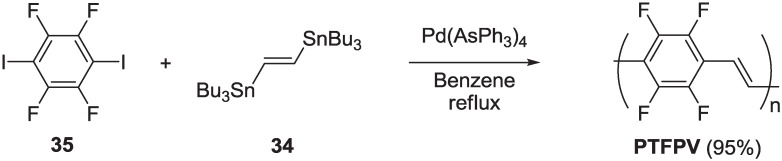
Synthesis of **PTFPV**.

Polyarylenevinylenes (PAVs) represent one of the most interesting and investigated classes of conjugated polymers for applications in optoelectronics devices, since Friend and coworkers demonstrated their use as emitting materials in organic polymer-based light emitting diodes [37]. A number of synthetic methods have been developed to prepare a wide variety of polyarylenevinylene derivatives showing excellent performance in several optoelectronic applications. Among these, the “precursor routes” (Gilch [38] or Wessling [39]) are versatile protocols and can be scaled up to a large scale production. They are based on the synthesis of soluble non-conjugated precursors that are converted in the final conjugated polymers after the elimination processes. Other classical methods are based on the Wittig [40,41,42] reaction and Knoevenagel [43,44,45,46] condensation. However, these methods do not allow accurate control of the stereochemistry of the vinylene units, which is of fundamental importance from the perspective of tailor electrical and optical properties of these materials for specific applications. Organometallic methods (*i.e.*, Heck, Suzuki-Miyaura, Stille) are very attractive from this point of view, thanks to their intrinsic high regio- and stereo-selectivity, which permits a large number of conjugated arylenevinylene materials to be prepared, both oligomers and polymers, with a high degree of structural stereo- and regio-regularity. In particular, the Stille reaction is highly stereoselective and extremely versatile in the synthesis of PAVs, starting from (*E*)-1,2-bis(tributylstannyl)ethene **34**, an organostannane building block that can be prepared in a large amount as a *trans*-vinylene unit source, used in cross-coupling with a number of aryl bis-halides. We have widely exploited the Stille reaction as a general synthetic strategy to prepare PAVs with various functional groups [47,48,49,50], with special attention, in the framework of this review, to the fluoro-functionalization, both on the aromatic ring and/or the vinylene units. Our investigation started with the synthesis of a PPV fully fluorinated on the aromatic rings, namely poly(tetrafluorophenylenevinylene) **PTFPV**. Before our work, unsuccessful attempts to synthesize the same polymer via a soluble precursor route [51] and via a self-condensation of (*E*)-2-(pentafluoropheny) ethenyl lithium [52] were reported by Brooke and Mawson. In our work, the Stille reaction was successfully carried out by coupling (*E*)-1,2-bis(tributylstannyl)ethene **34** and 1,4-diiodo-2,3,5,6-tetrafluorobenzene **35**, in the presence of Pd(AsPh_3_)_4_ as the catalyst (generated *in situ* from Pd_2_dba_3_ and AsPh_3_) in benzene (Scheme 9) [53].

**Figure 4:**
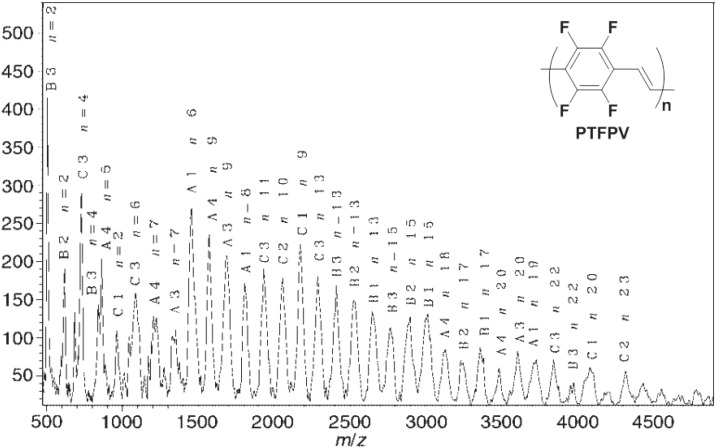
MALDI-TOF mass spectrum of **PTFPV**.

**Figure 5:**
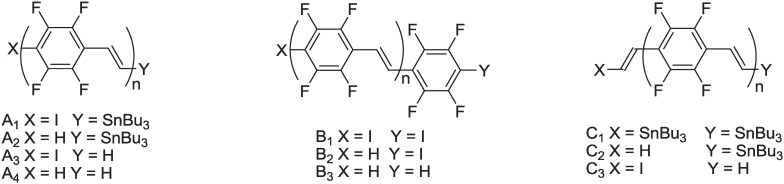
Polymeric chains of **PTFPV** detected in the MALDI-TOF mass spectrum.

The usual Pd(PPh_3_)_4_ resulted in being ineffective as the catalyst of the cross-coupling between reagents **34** and **35**, while substitution of the strong donor PPh_3_ ligand with the low donor AsPh_3_ ligand of palladium resulted in a very effective catalytic system. This experimental outcome is in according with data obtained in a previous study on the ligand effects in the Stille reaction [54]. In this study, the effect of changing the palladium ligands on the rates of a typical Stille cross-coupling (between iodobenzene and vinyltributyltin) was investigated, observing large rate enhancements associated with ligands of low donicity, like AsPh_3_ and tri(2-furyl)phosphine, compared to strong donors ligands, like the traditional PPh_3_. The pure polymer **PTFPV** was obtained in high yield as an insoluble yellow powder, after purification by washing the crude product of polymerization in a Soxhlet apparatus with hexane (24 h), methanol (24 h) and chloroform (12 h). It was characterized by Fourier transformed infrared spectroscopy (FTIR), X-ray photoelectron spectroscopy (XPS) and matrix assisted laser desorption ionization-time of flight (MALDI-TOF) mass spectrometry. Although the polymer was insoluble in common organic solvents, we were able to record its mass spectrum with the MALDI-TOF technique, using a protocol previously described for insoluble polyamides [55], which we extended for the first time to an insoluble conjugated polymer. The mass spectrum revealed peaks with masses ranging from 500 to 4500 amu, with average molecular weights of *M*w = 2400 and *M*n = 1700 and a polydispersity index of D > 1.4 (Figure 4). From the XPS characterization, assuming statistical chain terminations with one iodine atom and one tributylstannyl group, an average polymerization degree of approximately 17–20 arylenevinylene units can be deduced. The slight difference between the two methods may be attributed to the underestimated real molecular weights from the MALDI-TOF analysis, due to the mass discrimination phenomena at the higher molecular weights, which is intrinsic in this technique. The MALDI-TOF spectrum allowed also the correlation of the recorded peaks to **PTFPV** chains with all the possible chain terminations (Figure 5).

The FTIR confirmed the *trans*-configuration of vinylene units in the polymer by a characteristic strong band at 963 cm^−1^, due to the out of plane bending of the vinyl C–H bonds with *trans* geometry.

**Scheme 10:**
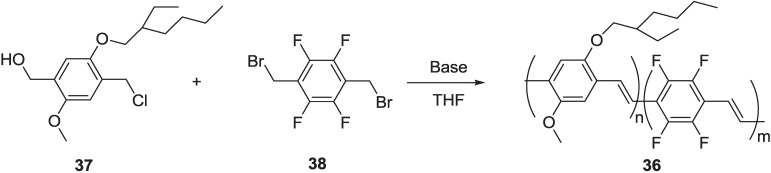
Synthesis of random copolymers **36** by Gilch-type polymerization.

It was demonstrated that the introduction onto the PPV skeleton of units with different electron affinity, such as alkoxy electron-donating and cyano electron-withdrawing groups, can be used as a valuable strategy to modify and adjust the HOMO and LUMO energy levels of the materials, obtaining a better matching with the work function of metal electrodes in OLED devices, thus improving performances [56,57]. In this contest, fluorine atoms appeared very interesting as for electron-withdrawing functionalities. Cacialli and coworkers [58] in 2000 reported the synthesis of random PPV copolymers, **36,** containing fluorine and alkoxy substituent, through the Gilch protocol starting from bis-(halomethyl) benzenes, **37** and **38** (Scheme 10).

**Scheme 11:**
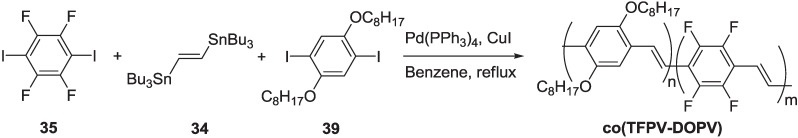
Synthesis of random copolymers **co(TFPV-DOPV)s** by Stille cross-coupling.

Varying the monomers’ feed ratios, three copolymers incorporating different fluorinated unit percentages, *i.e.*, 7%, 14% and 19% (weight ratios), respectively, were obtained. The loss of solubility of polymer chains when a higher percentage of fluorinated units was introduced prevented the synthesis of copolymers with a higher content of fluorinated units. By applying the Stille protocol, we were able to synthesize analogous random copolymers **co(TFPV-DOPV)s**, reacting (*E*)-1,2-bis(tributylstannyl)ethene **34** with variable ratios of the two aromatic monomers, 1,4-diiodo-2,3,5,6-tetrafluorobenzene **35** and 1,4-diiodo-2,5-bis(octyloxy)benzene **39** (Scheme 11) [59,60,61].

When an equimolar feed ratio of the two monomers, **35** and **39,** was used, the polymerization reaction resulted in a random copolymer **co(TFPV-DOPV)** (soluble in chlorinated solvents, such as CH_2_Cl_2_ and CHCl_3_) with a percentage of tetrafluorophenylene units as high as 63%, indicating a preferential incorporation of the fluorinated monomer (**35**), as a consequence of a higher reactivity of this reagent with respect to the alkoxy-substituted (**39**) one. The larger percentage of tetrafluorophenylene units in the random copolymer **co(TFPV-DOPV),** achievable with the Stille protocol with respect to the Gilch polymerization, can be attributed to a better solubility of the resulting polymers, due to the low molecular weight (*M*n = 2200 and *M*w = 2500, determined by MALDI-TOF mass analysis; *M*n = 2200 and *M*w = 3400, determined by size exclusion chromatography SEC).

#### 2.2.2. Optical and Electro-Optical Properties of Poly(arylenevinylene)s Fluorinated on the Aromatic Ring

**Figure 6:**
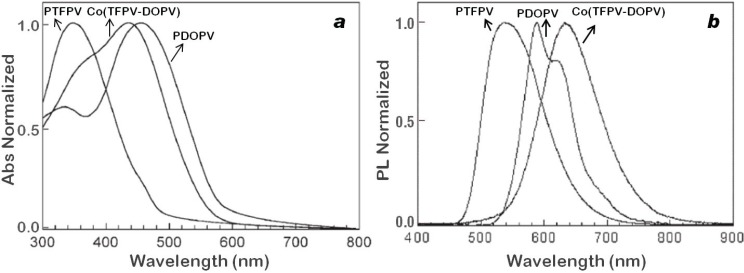
Normalized absorption (**a**) and emission (**b**) spectra of **PTFPV**, **PDOPV** and **co(TFPV-DOPV)** thin films.

In Figure 6, the normalized absorption (a) and emission (b) spectra in thin film of the copolymer **co(TFPV-DOPV)** are compared with those of the two related homopolymers, the fluorinated **PTFPV** and the dioctyloxy substituted **PDOPV**. Films of **PDOPV** and **co(TFPV-DOPV)** were spin coated from chloroform solution, while the **PTFPV** film was thermally evaporated under reduced pressure (10^−6^ mm Hg). The optical energy gap (Eg) estimated from the absorption spectra are, respectively, 2.95 eV for **PTFPV**, 2.32 eV for **PDOPV** and 2.51 eV for **co(TFPV-DOPV)**. The polymer **PDOPV** shows a maximum peak (HOMO-LUMO transition) at λ_max_ = 460 nm, while **PTFPV** shows a maximum peak at λ_max_ = 350 nm. The strong blue shift observed in the latter can be attributed to the inductive electron-withdrawing effect of the fluorine atoms on the aromatic rings [14,62], which is opposite to the electron-donating effect of the alkoxy substituents of **PDOPV**.

**Figure 7:**
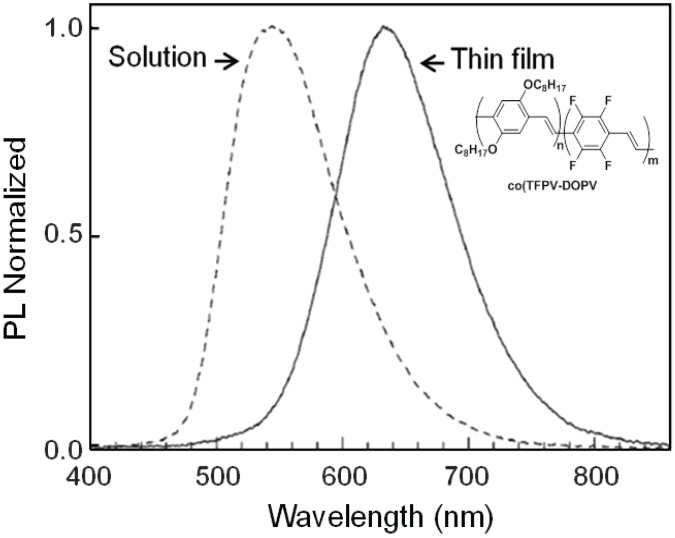
Emission spectra of **co(TFPV-DOPV)** in solution (dotted line) and thin film (solid line).

The copolymer, **co(TFPV-DOPV)**, shows an absorption spectrum with two maxima at 440 and 360 nm, which are close to the main absorption peaks of the two related homopolymers, thus suggesting a conjugated backbone containing two differently substituted polyphenylenevinylene segments, randomly arranged in the copolymer. The peak at 440 nm originates from chain segments containing the alkoxy substituted monomers, and it is slightly blue-shifted compared to the transition observed in pristine **PDOPV**, due to the presence of the electron-poor **PTFPV** segments. At the same time, the peak at 360 nm originates from **PTFPV** segments and is red-shifted with respect to the corresponding transition in pristine **PTFPV**, due to the presence of donor **PDOPV** segments. A different trend is observed in the emission spectra of the three polymers (Figure 6b); in fact, the emission maxima for **PDOPV**, **PTFPV** and **co(TFPV-DOPV)** are 580, 520 and 645 nm, respectively, with a significant red shift for the copolymer with respect to the homopolymers. All three polymers show emission spectra red-shifted with respect to the corresponding absorption spectra. In particular, **PDOPV** and **co(TFPV-DOPV)** show Stokes’ shifts of 110 and 190 nm, respectively. The higher Stokes shift observed for **co(TFPV-DOPV)** can be attributed to the more effective formation of interchain excited species in the solid state, due to the simultaneous presence of electron rich **DOPV** and electron poor **TFPV** segments. This consideration is supported by the broader and structureless shape of the **co(TFPV-DOPV)** emission spectrum in thin film. Moreover, for **co(TFPV-DOPV)**, the comparison of the emission spectra in solution and thin film (Figure 7) shows, as expected, a strong red-shift from solution to thin film, confirming the presence of exciton interchain migration, which is enhanced in the solid state.

Nonlinear optical properties (useful for applications in optical telecommunications) of **co(TFPV-DOPV)** and **PDOPV** have been investigated by measuring the third-order nonlinear susceptibility coefficient, χ^(3)^, in chloroform solution at λ = 1064 nm, with the picoseconds Z-scan technique [59,60,63]. A considerable improvement of |χ^(3)^|, exceeding one order of magnitude, was observed for **co(TFPV-DOPV)** (10^1^^0^ |χ^(3)^| ~ 6 esu) with respect to **PDOPV** (10^1^^0^ |χ^(3)^| ~ 0.5 esu). Measurements in thin film have been also performed by the Third Harmonic Generation technique in the infrared range between 1.2 and 2.1 mm, demonstrating an increased third order nonlinear optical coefficient of **co(TFPV-DOPV)** (10^11^ |χ^(3)^| ~ 4.3 esu) compared with those of the homopolymers **PDOPV** (10^11^ |χ^(3)^| ~ 1.2 esu) and **PTFPV** (10^11^ |χ^(3)^| ~ 1.2 esu). The enhancement of the nonlinear optical coefficients in **co(TFPV-DOPV)** compared to the corresponding homopolymers, **PDOPV** and **PTFPV**, can be attributed to the simultaneous presence of electron rich and electron poor units in the polymeric chains, which increase the polarizability of the whole conjugated system.

Finally, electrochemical and photoinduced spectroelectrochemical studies have been performed on **co(TFPV-DOPV)**, in order to investigate the nature of the species generated upon excitation and compare the photogenerated species with the electrochemical doping-induced charge carriers [64]. The result shows different infrared active vibration (IRAV) patterns for the electrochemical p- and n-doped **co(TFPV-DOPV)**, indicating the existence of different structures of positive and negative charge carriers during the electrochemical doping. Moreover, the photoinduced absorption IRAV patterns were found similar to the IRAV band patterns obtained during the electrochemical reduction (n-doping) and differ from that of electrochemical oxidation (p-doping). Such a behavior, observed for the first time in a conjugated polymer, makes **co(TFPV-DOPV)** an electrochemically both p- and n-dopable material, which may be important for the construction of optoelectronic devices, where photogeneration of negative charge carriers is required.

The electroluminescent properties of the fluorinated polymer **PTFPV** have been investigated in an ITO/TPD/PTFPV/Al OLED configuration, where the **PTFPV** thin film was deposited by thermal evaporation under reduced pressure (10^−6^ mm Hg) [53]. The presence of fluorine atoms assists the electron injection from the Al cathode, but raises the energy barrier to the hole injection from the ITO anode. In fact, the addition of a hole transporting layer (TPD) between the anode and the electroluminescent **PTFPV** layer was necessary to obtain a working device, characterized by green light emission and an electroluminescent threshold voltage of 6.5 V.

#### 2.2.3. Synthesis of Poly(arylenevinylene)s Fluorinated on the Vinylene Units

**Scheme 12:**
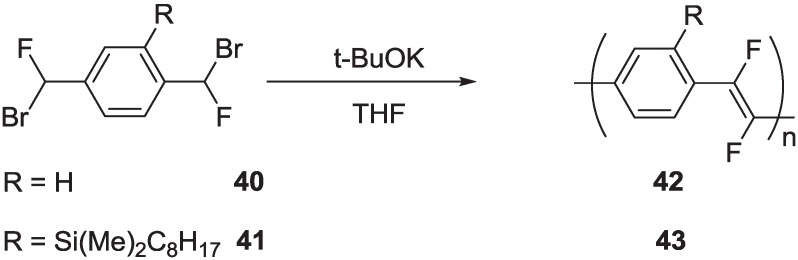
Synthesis of PPVs with fluorinated double bonds via the Gilch reaction.

Among the main drawbacks that limit commercial applications of PAVs in optoelectronic devices, the poor stability against the photodegradation processes, primarily localized on the double bonds, is certainly one of the most important. Overcoming this issue is necessary to increase the life-time of devices to match commercial requirements. To achieve this, substitution of C–H bonds in the vinylene units with stronger and less reactive C–F bonds can be used as a chemical modification of the conjugated backbone of PAVs able to increase their thermal chemical stability. In 2004, Su and coworkers [65,66] first reported the synthesis of two PPV polymers with fluorinated vinylene units via the Gilch reaction (Scheme 12).

**Scheme 13:**
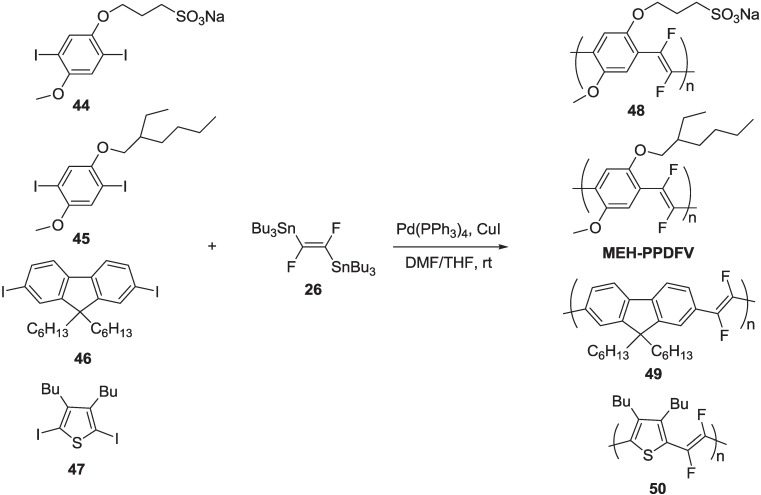
Synthesis of PAVs with fluorinated double bonds via the Stille cross-coupling.

Moreover, the Gilch protocol requires the use of strong bases, which limits the generality of this approach to the synthesis of PPVs with functional group that are base-insensitive [67]. We investigated the possibility of extending the Stille protocol to the synthesis of PAV polymers fluorinated on the vinylene units, taking advantage of the availability of (*E*)-1,2-difluoro-1,2-ethenediyl)bis(tributylstannane) **26** (Scheme 7), an organometallic reagent that can be used as a convenient source of a difluorovinyl building block in cross-coupling reactions. Then, we successfully reacted reagent **26** with various diiodoaryl derivatives **44**–**47**, as reported in Scheme 13 [68].

All polymerization was carried out at room temperature for one week, using Pd(PPh_3_)_4_ as the catalyst and CuI in a stoichiometric amount in DMF/THF. A final end-capping with phenyl tributyltin and iodobenzene is necessary for elimination of iodine and tributyltin terminal groups, respectively. Polymer **48** was purified by several crystallizations from DMF/acetone and obtained as a yellow-green powder. Polymers, **MEH-PPDFV** and **49**, were purified by Soxhlet extraction with hexane (24 h), methanol (24 h) and chloroform (12 h). The pure products were recovered from chloroform and obtained as green and light-green powders, respectively. Polymer **50** was purified by extraction in a Soxhlet apparatus with dichloromethane, followed by crystallization from dichloromethane/methanol (twice) and obtained as a red powder. The average molecular weights *M*n and *M*w of polymers **MEH-PPDFV**, **49** and **50** were determined by conventional size exclusion chromatography (SEC), while a multiangle light scattering (MALS) detector on line to a SEC system was used for polymer **48** (Table 2).

**Table 2:** Yields, molecular weights and polydispersivities of polymers **48**–**50** and **MEH-PPDFV**.

Polymer	Yield (%)	Mw	Mw/Mn
48	55	65150	1.6
MEH-PPDFV	87	47000	1.7
49	82	45600	2.0
50	50	47100	2.9

**Scheme 14:**
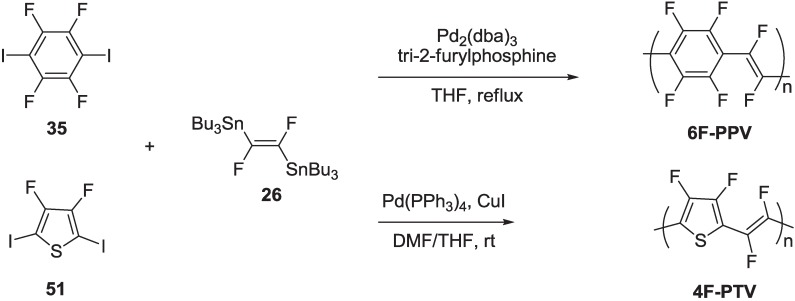
Synthesis of polymers **6F-PPV** and **4F-PTV**.

The Stille protocol was also extended to the synthesis of two new perfluorinated poly(arylenevinylene)s, namely poly(1,4-tetrafluorophenylenedifluorovinylene) **6F-PPV** and poly(2,5-difluorothienylenedifluorovinylene) **4F-PTV**, starting from organostannane **26** coupled with the diiodo perfluorinated aryl compounds **35** and **51**, respectively (Scheme 14) [69].

Polymer **4F-PTV** was synthesized using the same catalytic system employed for the preparation of polymers **48**–**50** and **MEH-PPDFV**, while polymer **6F-PPV** required a different catalyst, generated *in situ* from Pd_2_(dba)_3_ and tri-2-furylphosphine, in refluxing THF. Both the polymers **6F-PPV** and **4F-PTV** were purified by washing the crude product of polymerization in a Soxhlet apparatus with hexane (24 h), methanol (24 h) and chloroform (12 h) and were collected as insoluble (in common organic and perfluorinated solvents) green and red powders, respectively. They were characterized by FT-IR and MALDI-TOF mass spectrometry. The IR spectra show two significant peaks due to the C–F stretching vibration of vinylene units (1165 cm^−1^ for **4F-PTV** and 1173 cm^−1^ for **6F-PPV**) and the C–F stretching vibration of aromatic rings (1014 cm^−1^ for **4F-PTV** and 989 cm^−1^ for **6F-PPV**). The MALDI-TOF analysis reveals a polymerization length of 3–9 repeating units for **6F-PPV** (*M*n = 1330, *M*w = 1400; polydispersity index, D ~ 1.05) and 6–12 for **4F-PTV** (*M*n = 1690, *M*w = 1750; polydispersity index, D ~ 1.04). Despite the mass discrimination phenomena in favor of low molecular weight chains, typical of the MALDI-TOF analysis, taking in account the low solubility of the growing polymer chains of these perfluorinated PAVs, we consider the molecular weights determined by MALDI-TOF mass spectra a good approximation of the real mass distribution of the final polymers. On the other hand, low molecular weight does not represent a limit to most applications of conjugated polymers as semiconductors, since also oligomers can exhibit optical and electrical properties suitable for device applications, with the additional advantage of using even the thermal evaporation-deposition as a processing method. This is, in fact, the case for polymers **6F-PPV** and **4F-PTV**, which can be thermally evaporated in good quality thin films under reduced pressure (about 10^−5^ mbar).

**Scheme 15:**
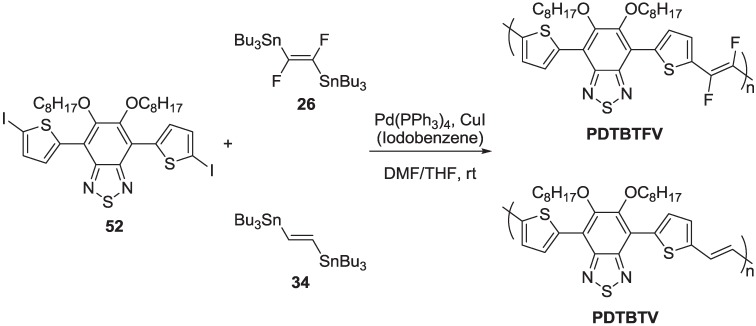
Synthesis of polymers **PDTBTFV** and **PDTBTV**.

More recently, we focused our attention on the potentiality of fluorinated arylenevinylene polymers suitable for photovoltaic applications, as donor materials in bulk heterojunction (BHJ) solar cells. Some interesting papers [70,71,72,73] refer to favorable effects of the fluorine atoms on the performance of polymers used as donor materials in blend with phenyl-C61-butyric acid methyl ester (PCBM) derivatives in BHJ solar cells. The role of the fluorine is mainly attributed to its capability of lowering both the HOMO and LUMO energy levels of the donor, which causes an increase in the open circuit voltage, V_OC_. Moreover, the increase in the thermal and chemical stability induced by the fluoro-functionalization of the organic materials makes this little and powerful substituent an intriguing tool to achieve organic-based long-lifetime devices. In search of new low band gap materials suitable for organic photovoltaics, the introduction of vinylene units in the conjugated backbone of polymers has been proposed as a structural modification to reduce the band gap, as in PPVs [74,75,76,77,78,79,80,81,82,83] and poly(thienylenevinylene)s (PTVs) [84,85,86,87,88]. In fact, several studies have reported on the effect of double bonds in donor polymers for BHJ solar cells, especially as a bridge between donor and acceptor units in donor-acceptor type low band gap polymers [89,90,91,92,93,94,95,96,97,98]. In our investigation, we focused our attention on the class of PAVs, with the main aim to study the effects of fluoro-functionalization of double bonds, in a molecular structure, which is characterized by a low band gap of the final polymer. Whit this aim, we designed and synthesized a PAV alternating fluorinated double bond and bis-thienyl-bis-alkoxy-benzothiadiazole units **(PDTBTFV)**, together with its non-fluorinated counterpart (**PDTBTV)**, starting from organostannane **26** and **34** coupled with diiodo derivative **52** in the Stille reaction conditions (Scheme 15) [99].

The polymers were purified by washing the crude product of polymerization in a Soxhlet apparatus with hexane (24 h) and methanol (24 h). A final extraction with chloroform yields the pure polymers as dark violet (**PDTBTV**) and dark blue (**PDTBTFV**) powder, in good yield. The average molecular weight was determined by size exclusion chromatography (SEC). The results are reported in Table 3.

**Table 3:** Yields, molecular weights and polydispersivities of polymers **PDTBTV** and **PDTBTFV**.

Polymer	Yield (%)	*M*w	*M*w/*M*n
PDTBTV	70	36,900	4.67
PDTBTFV	75	43,700	4.46

Both the polymers are soluble in chlorinated solvents, such as chloroform, chlorobenzene (CB) and *ortho*-dichlorobenzene (ODCB).

#### 2.2.4. Optical and Electro-Optical Properties of Poly(arylenevinylene)s Fluorinated on the Vinylene Units

**Figure 8:**
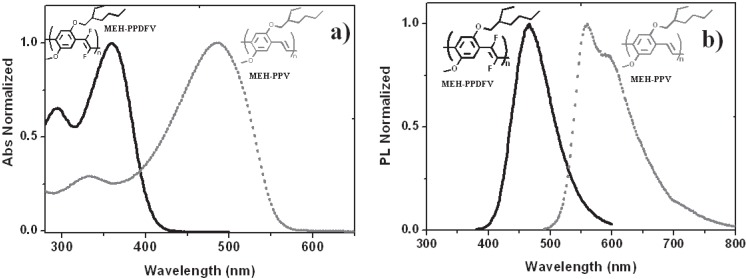
Normalized absorption (**a**) and emission (**b**) spectra in chloroform solution of **MEH-PPDFV** and **MEH-PPV**.

In order to study the effects of fluoro-functionalization of the double bond in alkoxy-substituted poly(arylenevinylene)s, we compared the spectroscopic properties of the fluorinated **MEH-PPDFV** with those of the non-fluorinated analogous, **MEH-PPV**. Fluorination of double bonds causes a strong hypsochromic shift of the absorption and emission spectra, both in solution and solid state. In fact, in chloroform solution, **MEH-PPDFV** showed a blue shift of 120 nm of the absorption maximum (λ_max_^abs^ = 360 nm) with respect to **MEH-PPV** (λ_max_^abs^ = 480 nm) and a corresponding blue shift of 87 nm was observed in the PL spectra (λ_max_^PL^ = 468 nm for **MEH-PPDFV** and λ_max_^PL^ = 555 nm for **MEH-PPV**) (Figure 8).

**Figure 9:**
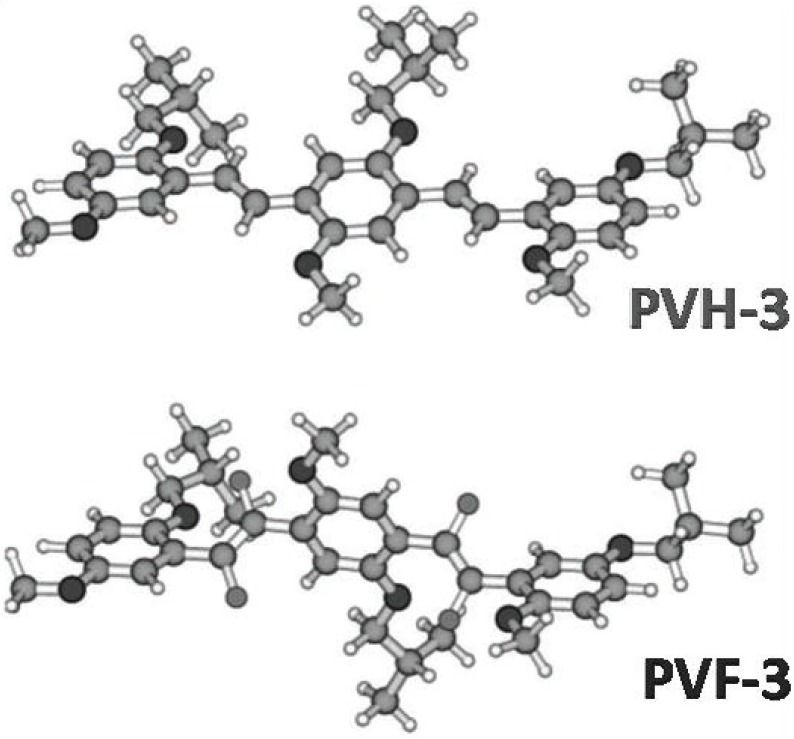
**BH-LYP/TZVP** optimized geometries of the model oligomers, **PVF-3** and **PVH-3**. Reproduced with permission from [34]. Copyright 2008 the American Chemical Society.

Theoretical calculations on model oligomers (**PVF-3,** the fluorinated, and **PVH-3,** the non-fluorinated; Figure 9) performed by the Time Dependent Density Functional Theory (TD-DFT) [34] assign the large blue shift induced by the fluorination of the double bond mainly to the steric repulsion between the fluorine atoms on the vinylene units and the oxygen atoms of the alkoxy substituents on the neighboring phenylene units, rather than to the electron-withdrawing effect of the fluorine. The theoretical predictions were experimentally confirmed by the Raman spectroscopy measurements [100].

**Figure 10:**
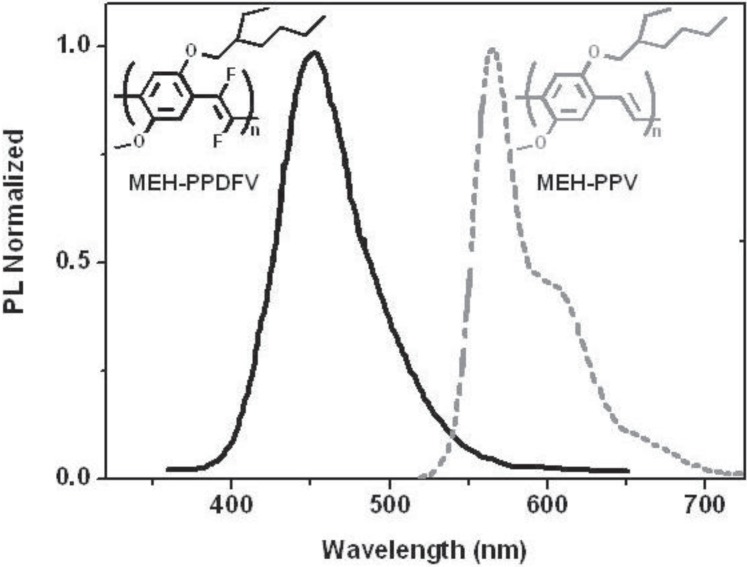
PL spectra of thin films of **MEH-PPV** and **MEH-PPDFV**.

Through spectroscopic ellipsometry, the solid state optical properties of **MEH-PPDFV** and **MEH-PPV** have been investigated [101,102]. Thin films of **MEH-PPDFV** show an absorption maximum up to about 3.7 eV (~330 nm), the highest value reported so far for a poly(*p*-phenylenevinylene) polymer, and it is up to 30 nm blue shifted from the value measured for the same polymer in chloroform solution (λ_max_^abs^ = 360 nm). Similarly, thin films of **MEH-PPDFV** show a strong blue photoluminescence in room temperature, with the emission maximum at 458 nm, which is blue shifted of 10 nm with respect to the photoluminescence in solution (468 nm). This behavior is opposite to that observed for the polymer, **MEH-PPV**, and in general, for most conjugated polymers, where a red shift from solution to thin film, both for the emission and absorption peaks, is observed. The blue shift of the emission spectrum moving from solution to the solid state indicates that no interchain aggregation occurs for **MEH-PPDFV** in thin film. Moreover, in the solid state, the emission maximum of **MEH-PPDFV** is blue-shifted about 110 nm with respect to that of **MEH-PPV** (λ_max_ = 568 nm), synthesized by the same methodology (Figure 10), resulting in the most blue shifted emission reported so far for a poly(*p*-phenylenevinylene) polymer.

**Figure 11:**
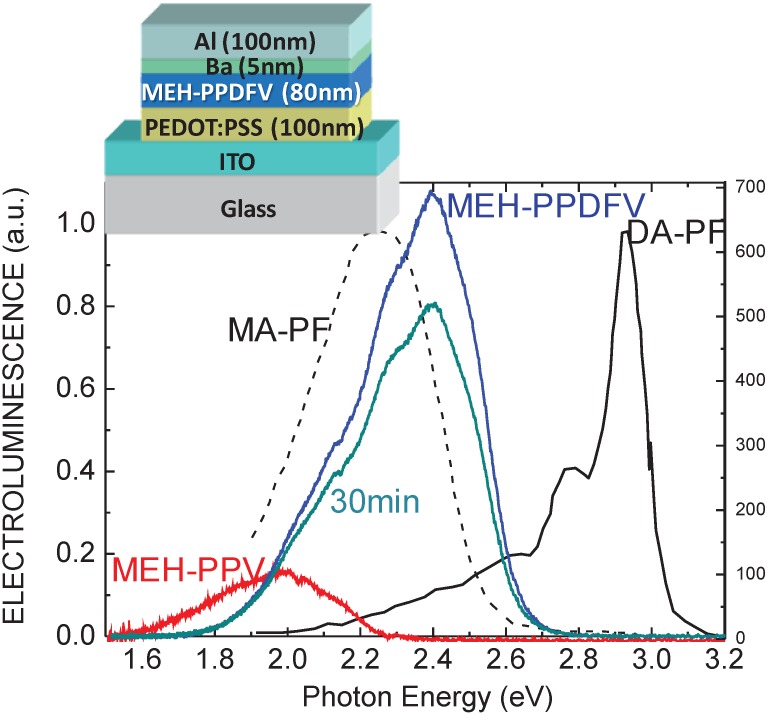
EL spectra of 80 nm thick films of **MEH-PPDFV** (blue curves) and **MEH-PPV** (red curves) recorded under identical experimental conditions using the device structure shown in the scheme in the inset. The **MEH-PPDFV** spectrum obtained after 30 min (dark green curve) of continuous operation is also shown. For comparison, the normalized EL spectra of an ITO/MA-PF/Al and of an ITO/DA-PF/Al [103] (left vertical axis) are also shown, where **MA-PF** indicates a 9-monoalkylated polyfluorene and **DA-PF** indicates a 9,9-dialkyl polyfluorene. Reproduced with permission from [102]. Copyright 2009 Wiley-VCH Verlag GmbH & Co. KGaA.

The electroluminescent properties of **MEH-PPDFV** have been investigated in comparison with those of **MEH-PPV** in a ITO/PEDOT:PSS/MEH-PPDFV(MEH-PPV)/Ba/Al OLED device (Figure 11 [103]). The EL spectra of **MEH-PPDFV** and **MEH-PPV** exhibit the maximum of peaks at 2.46 eV (504 nm) and 1.98 eV (625 nm), respectively, which correspond to blue-greenish and red emission, showing a blue-shift of 120 nm for the fluorinated polymer. A strong increase in the EL intensity of **MEH-PPDFV** compared to that of the non-fluorinated **MEH-PPV** is also evident, indicating a higher degree of twisting in the conjugated backbone caused by the F atoms, in agreement with theoretical predictions [34], which should reduce the interchain interactions in the solid state, thus increasing the EL efficiency. The EL of **MEH-PPDFV** is also quite stable, and after 30 min of continuous operation in air, only a slight decrease in intensity with no red-shift was observed [102].

It is worth noting that **MEH-PPDFV** shows a blue-shift in the absorption and comparable PL emission wavelength with respect to **DA-PF**, which is one of the most representative blue emitting conjugated polymers exhibiting a π–π* transition peak at 3.23 eV (384 nm) and a PL maximum ranging between 2.85 (436 nm) [104] and 2.7 eV (460 nm) [105]. On the contrary, the blue-greenish electroluminescent peak of **MEH-PPDFV** at 2.46 eV is red shifted with respect to that of **DA-PF** at 2.9 eV, but still blue-shifted with respect to that of **MA-PF** at 2.25 eV [103] (Figure 11).

The optical properties of the perfluorinated polymers, **6F-PPV** and **4F-PTV**, in the solid state have also been investigated by spectroscopic ellipsometry [69]. Thin films of **6F-PPV** and **4F-PTV** were obtained by thermal evaporation under reduced pressure. The optical band gap, E_g_^opt^, determined from the onset of absorption was found at 3.34 eV for **6F-PPV** and 2.07 eV for **4F-PTV**, while the maximum of the absorption, which corresponds to the HOMO-LUMO transition, was found at 4.18 eV and 2.55 eV, respectively. By comparing the band gap values of the polymers, **6F-PPV** and **4F-PTV**, with those of the corresponding non-fluorinated polymers, the poly(1,4-phenylenevinylene) (**PPV**, band gap = 2.4 eV [106]) and poly(2,5-thienylenevinylene) (**PTV**, band gap = 1.74 eV [87]), a strong blue-shift of the optical band gap due to perfluorination can be observed, with the shift being more pronounced for **6F-PPV** than for **4F-PTV**. Interestingly, the polymer, **6F-PPV**, shows a band gap value even higher than that of **MEH-PPDFV**. The reason of the strong blue-shift observed for **6F-PPV** and **4F-PTV** may reside in several structural factors, such as reduced polymer chain length, the strong electron-withdrawing effect of fluorine atoms and steric repulsion between fluorine atoms on vinylene units and fluorine atoms *ortho-* to vinylene units on aromatic rings.

#### 2.2.5. Photovoltaic Performance of **PDTBTFV** and **PDTBTV** in BHJ Solar Cells: Effects of Fluorination of Double Bonds

**PDTBTFV** and **PDTBTV** were used as donor materials in blend with PCBM in BHJ solar cells. Table 4 reports the photovoltaic performance recorded on non-optimized devices [99].

**Figure 12:**
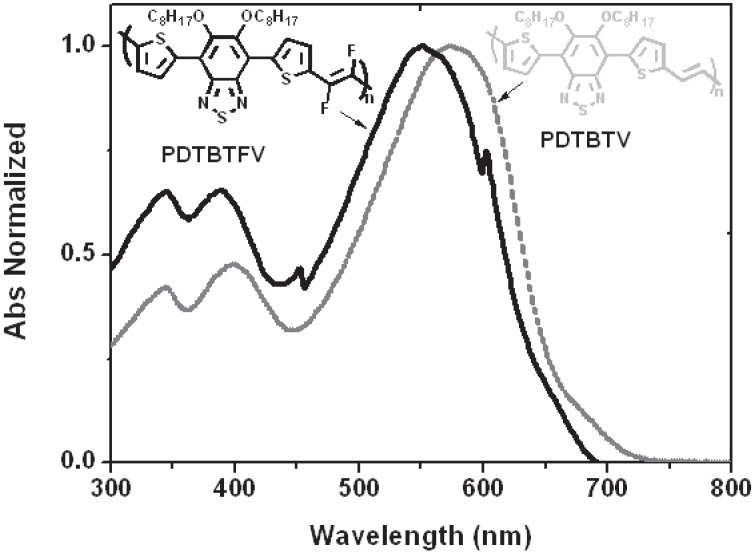
Normalized absorption spectra in chloroform solution of polymers **PDTBTFV** and **PDTBTV**.

The devices fabricated with the fluorinated polymer **PDTBTFV** show, in general, better photovoltaic performance with significantly higher V_OC_ and η values compared to the non-fluorinated **PDTBTV** for all the measured weight ratios. Thermal annealing of the blend in the range 70–150 °C does not significantly affect the photovoltaic performances of both polymers, according to the data of the differential scanning calorimetry (DSC) analysis, which reveals the lack of any transition. Theoretical, electrochemical and optical characterization have been carried out in order to shed light on the interesting achievement of the capability of fluorine to enhance the photovoltaic properties of the polyconjugated structure investigated. The absorption spectra in chloroform solution of the two polymers **PDTBTFV** and **PDTBTV** are reported in Figure 12.

**Table 4:** Photovoltaic performance of BHJ solar cells of **PDTBTFV** and **PDTBTV** in blend with PCBM: open circuit voltage, V_OC_; short circuit current, I_SC_; fill factor FF and power conversion efficiency, η.

Polymer	Polymer/PCBM Weight ratio	V_OC_ [V]	I_SC_ [mA cm^−2^]	FF [%]	η [%]
PDTBTFV	2:7	0.73	2.94	38.57	0.83
	3:7	0.75	3.55	37.97	1.05
	1:1	0.83	4.32	34.56	1.24
PDTBTV	2:7	0.55	3.33	28.83	0.53
	3:7	0.56	2.42	24.50	0.33
	1:1	0.43	3.15	33.51	0.46

**Figure 13:**
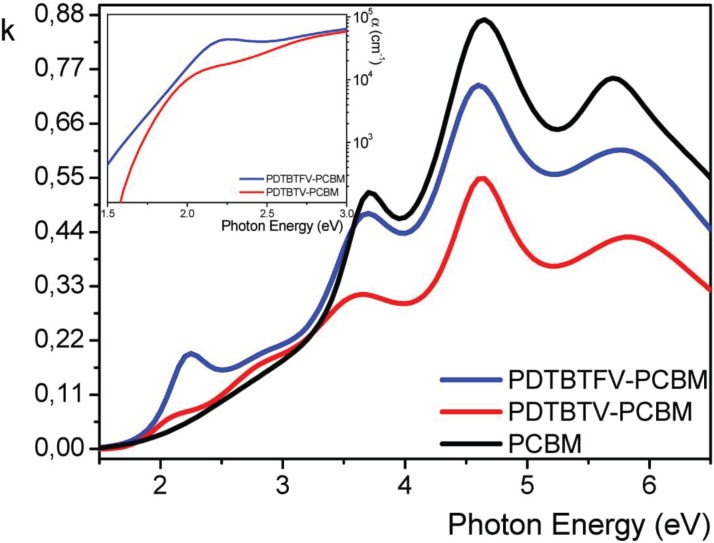
Ellipsometric spectra of the extinction coefficient and, in the inset, of the absorption coefficient for the fluorinated **PDTBTFV** and non-fluorinated **PDTBTV** polymers blended with phenyl-C61-butyric acid methyl ester (PCBM) (polymer/PCBM ratio 1:1). For comparison, the spectrum of PCBM is also shown.

Both polymers show very wide absorption in the visible region, with maxima determined at 548 nm (2.26 eV) for **PDTBTFV** and 569 nm (2.18 eV) for **PDTBTV**, resulting in a fluorination-induced blue-shift of 0.08 eV. Such blue-shift can be ascribed to the interplay of the mesomeric and inductive effect of fluorine atoms, with the latter effect dominating in the conjugated backbone of the investigate polymer structure, according to the results of theoretical TD-DFT calculations. The optical properties in the solid state have been investigated by spectroscopic ellipsometry, revealing the same trend in the absorption spectrum found in solution, with the optical band gap of the films determined at 1.79 eV for **PDTBTFV** and 1.69 eV for **PDTBTV**. In cyclic voltammetry, the oxidation and reduction processes have been investigated and the HOMO and LUMO energy levels estimated (based on Koopmans’ theorem). The estimated HOMO energy levels are −5.20 eV for **PDTBTFV** and −5.01 eV for **PDTBTV**, while the LUMO energy levels are −3.14 for **PDTBTFV** and −3.03 for **PDTBTV**. Hence, the fluoro-functionalization of vinylene units results in an overall stabilization of both HOMO and LUMO energy levels, in agreement with data obtained from DFT calculations. The ellipsometric characterization discloses also a new remarkable effect of fluoro-functionalization of the double bonds, which is a significant enhancement of thin film light absorption in **PDTBTFV** compared to **PDTBTV**. This result can be attributed to a different packing of polymer chains in the solid state, with the fluorination of vinylene units being responsible for the increased intermolecular interactions between almost coplanar adjacent polymer chains, which, depending on the nature of the chains and interactions, can result in the observed increased absorption coefficient. When the polymers are blended with PCBM for the fabrication of BHJ solar cells, the absorption coefficient of the **PDTBTFV**-PCBM blend still remains higher than that of the **PDTBTV**-PCBM blend in the region of the main optical absorption of the polymer (Figure 13).

This enhancement of light absorption of the fluorinated polymer might be a main reason for the better photovoltaic performance of **PDTBTFV** compared to **PDTBTV**, since a higher number of free charge carriers is expected to be generated as a consequence of the higher light absorption. Summing up, the favorable effect of double bond fluorination on the photovoltaic performance of the poly(arylenevinylene) structure investigated can be attributed to a synergetic effect of (i) the lowering of HOMO and LUMO energy levels, leading to an increase in V_OC_, and (ii) the increase in the molar extinction coefficient. The latter is very attractive from the perspective of reduction of BHJ film thickness, which should enhance the charge carrier generation at the interface and enables collection of carriers at the electrodes against their tendency to recombine.

## 3. Conclusions

In summary, we have presented a series of arylenevinylene compounds, synthesized through the Stille cross-coupling reaction, selectively fluoro-functionalized on the aromatic ring and/or the vinylene units. Both oligomeric and polymeric materials have been prepared with a rigorous control of the conjugated molecular structure by an appropriate choice of monomers and experimental reaction conditions. The optical and electro-optical properties of the synthesized materials have been investigated in order to highlight the impact of fluorination on the properties, revealing electronic and steric effects that deeply affect the characteristics of the conjugated system. In many cases, fluorinated materials disclose enhanced properties with respect to the corresponding non-fluorinated materials, and better performance in electro-optical devices has been obtained. In particular, the fluorination of double bonds in PPVs with alkoxy substituents on the aromatic rings causes a strong blue shift of the optical properties, resulting in a new kind of blue-emitting polymers with enhanced EL and device stability. Simultaneously, the fluorination of double bonds in a PAV structure with low band gap significantly increases the light absorption capability in the solid state and, as a consequence, the photovoltaic performance in BHJ solar cells. The results referred to here show a special uncommon capability of fluorine to modulate optical and electrical properties of polyconjugated materials, offering a powerful tool in the hands of synthetic chemists for tailoring the properties of materials for specific applications.
